# ISMB 2016 offers outstanding science, networking, and celebration

**DOI:** 10.12688/f1000research.8640.1

**Published:** 2016-06-14

**Authors:** Christiana Fogg

**Affiliations:** 1 International Society for Computational Biology, La Jolla, CA, USA

**Keywords:** computational biology, molecular biology, statistics

## Abstract

The annual international conference on Intelligent Systems for Molecular Biology (ISMB) is the major meeting of the International Society for Computational Biology (ISCB). Over the past 23 years the ISMB conference has grown to become the world's largest bioinformatics/computational biology conference. ISMB 2016 will be the year's most important computational biology event globally.

The conferences provide a multidisciplinary forum for disseminating the latest developments in bioinformatics/computational biology. ISMB brings together scientists from computer science, molecular biology, mathematics, statistics and related fields. Its principal focus is on the development and application of advanced computational methods for biological problems. ISMB 2016 offers the strongest scientific program and the broadest scope of any international bioinformatics/computational biology conference. Building on past successes, the conference is designed to cater to variety of disciplines within the bioinformatics/computational biology community.

ISMB 2016 takes place July 8 - 12 at the Swan and Dolphin Hotel in Orlando, Florida, United States. For two days preceding the conference, additional opportunities including Satellite Meetings, Student Council Symposium, and a selection of Special Interest Group Meetings and Applied Knowledge Exchange Sessions (AKES) are all offered to enable registered participants to learn more on the latest methods and tools within specialty research areas.

Orlando, Florida is home to this year’s ISMB (Intelligent Systems for Molecular Biology) meeting, which will feature a world-class program of talks, poster sessions, and opportunities for scientific exchanges. ISMB is the flagship conference of ISCB and is renowned for bringing together scientists from diverse disciplines, including molecular biology, biophysics, medicine, computer science, mathematics and statistics. The ISMB program showcases cutting edge research that spans the realm of computational biology from algorithm development to applications in medicine and agriculture. This year’s meeting will take place from July 10 – 12, 2016 at the Swan and Dolphin Hotel in Orlando, Florida and will be preceded by satellite meetings, special interest group (SIG) meetings, and applied knowledge exchange sessions (AKES) on July 8th and 9th.

Pierre Baldi of the University of California, Irvine and Teresa Przytycka of the National Center for Biotechnology Information at the National Library of Medicine, NIH are co-chairs of ISMB 2016. Baldi said, “ISMB remains the premier international conference in bioinformatics. ISMB 2016 has an outstanding technical program of invited and accepted presentations, awards, and other activities. In addition to scientific excellence, I am also personally impressed by the outreach activities of our society and how it strives for inclusiveness and broad participation. As an example, this year eight women scientist were elevated to the title of ISCB Fellow.” Przytycka added, “An important aspect of the conference is that presentations often describe computational solutions utilizing cutting edge technological advances in experimental science.”

Highlights, late-breaking research, proceedings and posters will be categorized thematically under the data, disease, genes, proteins, or networks theme areas. This organizational approach was launched successfully at ISMB/ECCB 2015 and enabled conference attendees to identify and attend more relevant talks. This organization also streamlined the conference schedule to reduce the number of concurrent sessions.

As in past conferences, ISMB is anchored by six keynote presentations delivered by leaders in the fields of computational biology and bioinformatics. Ruth Nussinov of the National Cancer Institute/National Institutes of Health is the ISCB Fellows speaker and will open up the conference with her keynote presentation on the morning of Sunday, July 10. This year’s Overton Prize winner, Debora Marks of Harvard Medical School, will give a keynote presentation on Sunday afternoon in recognition of her award. Monday, July 11 will include keynote presentations by Sandrine Dudoit of the University of California, Berkeley and Sarah Teichmann of the EMBL-EBI and Wellcome Trust Sanger Institute. Keynote presentations on July 12, the final day of the meeting, will showcase the recipients of the inaugural ISCB Innovator award Serafim Batzoglou of Stanford University and the Accomplishment by a Senior Scientist Award winner Søren Brunak of the Novo Nordisk Foundation Center for Protein Research, University of Copenhagen, Denmark.

Several special sessions have been organized for ISMB 2016 and highlight topics of growing interest for computational biologists, including ribosome profiling, data compression, tertiary data analysis, and molecular communication and nano-networking with applications to precision medicine. The Industry Advisory Council (IAC) is hosting a Special Industry Session called “Industrial Biotechnologies - Opportunities and Challenges.” This session will showcase computational biology opportunities and challenges in industry, and will include industry representatives discussing areas such as protein production, vaccine development and biofuels.

Several Birds of a Feather (BoF) sessions are being organized during ISMB 2016 including Navigating the Career Path: Industry, Diversity and Science, Open Science Publishing, and Exploring and Refining Core Competencies in Bioinformatics.

The Career Fair @ ISMB is making a return! ISCB members will have access to an online jobs board and will be able post CVs and resumes through the ISCB Career Center. Stay tuned for news about employers and recruiters that will be onsite during ISMB.

The annual ISCB open business meeting will be held at ISMB 2016 under its new title of ISCB Town Hall. This event is open to any attendee and is a great opportunity for ISCB members to hear first hand about the state of the Society and programs, and includes an awards ceremony for Student Council Symposium winners and a question and answer period to help shape the direction and scope of ISCB.

The exhibit hall will be a hub of activity for attendees throughout the conference where they can learn about cutting edge technologies, resources, and products from exhibitors and take in poster sessions during the early evenings. Exhibitors will be presenting their latest developments in technology track presentations throughout the meeting as well. The ISMB Art & Science Exhibition will also be on display in the exhibit hall and will offer unique perspectives of scientific content.

Register now ( www.iscb.org/ismb2016) to make sure you are part of this world class computational biology meeting! And because ISMB 2016 is meeting in “the happiest place on Earth,” check out ISMB-associated discounts for access its world famous amusement parks.

